# A hidden Markov model for haplotype inference for present-absent data of clustered genes using identified haplotypes and haplotype patterns

**DOI:** 10.3389/fgene.2014.00267

**Published:** 2014-08-12

**Authors:** Jihua Wu, Guo-Bo Chen, Degui Zhi, Nianjun Liu, Kui Zhang

**Affiliations:** ^1^Section on Statistical Genetics, Department of Biostatistics, University of Alabama at BirminghamBirmingham, AL, USA; ^2^Queensland Brain Institute, The University of QueenslandSt. Lucia, QLD, Australia

**Keywords:** Hidden Markov model, haplotype, haplotype inference, KIR genes, haplotype patterns

## Abstract

The majority of killer cell immunoglobin-like receptor (KIR) genes are detected as either present or absent using locus-specific genotyping technology. Ambiguity arises from the presence of a specific KIR gene since the exact copy number (one or two) of that gene is unknown. Therefore, haplotype inference for these genes is becoming more challenging due to such large portion of missing information. Meantime, many haplotypes and partial haplotype patterns have been previously identified due to tight linkage disequilibrium (LD) among these clustered genes thus can be incorporated to facilitate haplotype inference. In this paper, we developed a hidden Markov model (HMM) based method that can incorporate identified haplotypes or partial haplotype patterns for haplotype inference from present-absent data of clustered genes (e.g., KIR genes). We compared its performance with an expectation maximization (EM) based method previously developed in terms of haplotype assignments and haplotype frequency estimation through extensive simulations for KIR genes. The simulation results showed that the new HMM based method outperformed the previous method when some incorrect haplotypes were included as identified haplotypes and/or the standard deviation of haplotype frequencies were small. We also compared the performance of our method with two methods that do not use previously identified haplotypes and haplotype patterns, including an EM based method, HPALORE, and a HMM based method, MaCH. Our simulation results showed that the incorporation of identified haplotypes and partial haplotype patterns can improve accuracy for haplotype inference. The new software package HaploHMM is available and can be downloaded at http://www.soph.uab.edu/ssg/files/People/KZhang/HaploHMM/haplohmm-index.html.

## Introduction

Population-based association studies including both genome-wide mapping and fine mapping of complex disease genes have become increasingly popular as they offer a potentially more cost-effective and powerful approach than linkage analysis (Ardlie et al., 2002; Botstein and Risch, 2003). The unphased genotype data that are available from most of association studies can be analyzed based on single markers, multiple markers, or haplotypes. Haplotype based analysis can provide additional power in defining effects associated with multiple disease-related alleles within a single gene (Morris and Kaplan, 2002) or when a single marker test fails to capture local complexity of linkage disequilibrium (LD) between multiple markers (Akey et al., 2001). For some diseases such as hypertension, rare haplotypes have been shown to influence the disease susceptibility (Liu et al., 2005; Zhu et al., 2005; Kitsios and Zintzaras, 2010). In addition, haplotype information is crucial to better understanding of human linkage disequilibrium (LD) patterns, impute untyped genetic variants (Marchini et al., 2007), and infer human population history (Tishkoff et al., 1996; Liu et al., 2004).

Practically, haplotypes within individuals of a set of study samples can be experimentally obtained through laboratory techniques such as long-range PCR or chromosomal isolation (e.g., Michalatos-Beloin et al., 1996; Yan et al., 2000; Douglas et al., 2001), but these approaches are often too expensive and too cumbersome to be used effectively for large-scale studies. Therefore, the vast amount of data generated from most of association studies is still mainly unphased genotypes. For such data, we need to rely on statistical and computational methods to infer haplotypes through estimation of haplotype frequencies and assignment of haplotype pairs (diplotypes) within individuals. Accordingly, effective and accurate methods for haplotype inference in various situations are quite valuable.

Many methods for haplotype inference for genotypes have been developed in recent years (e.g., Excoffier and Slatkin, 1995; Hawley and Kidd, 1995; Stephens et al., 2001; Niu et al., 2002; Qin et al., 2002; Zhang et al., 2005; Liu et al., 2006; Yoo et al., 2007). Recently, more advanced methods based on Hidden Markov Model have been developed and shown more accurate (Stephens and Scheet, 2005; Scheet and Stephens, 2006; Li et al., 2010). Theoretically, aforementioned methods for haplotype inference for genotypes at SNP loci can be extended for genotypes from copy number variations. At the same time, several methods have been developed for haplotype inference for copy number variations (Su et al., 2010; Kato et al., 2011; Ho Jang et al., 2013). However, most of them haven't been specifically tailored for analyses of the Killer immunoglobulin-like receptor (KIR) gene family as detected with locus-specific technology.

The human Killer-cell immmunoglobin-like receptor (KIR) gene family is a cluster of genes located in a highly polymorphic region at the chromosome 19q13.4 (Hsu et al., 2002; Martin et al., 2004; Middleton et al., 2005). KIR genes encode receptors in the membrane of natural killer (NK) cells which are known to regulate the killing function of NK cells by interacting with major histocompatibility complex (MHC) class I molecules but the exact mechanisms are not fully understood (Middleton et al., 2005). To date, 17 genes and pseudogenes have been assigned to the KIR gene family (Marsh et al., 2003). Discovery of allelic variants within each KIR gene is still in the early stage (Hsu et al., 2002), so current KIR genotyping is almost exclusively restricted to locus-specific PCR, which detects the presence or absence of target genes. Therefore, the exact number of copy of gene (one or two) is not determined when that gene is detected as the presence (Hsu et al., 2002; Middleton et al., 2005), resulting in limited genotype data. For such present-absent genotype data of KIR genes, haplotypes defining gene contents could be inferred using some simple logic rules if there is enough familial information for each individual (Hsu et al., 2002; Middleton et al., 2005). Studies so far have revealed 42 different haplotypes involving different combinations of 17 KIR genes (Khakoo and Carrington, 2006), including four—3DL3, 3DP1, 2DL4, and 3DL2—that are found on all KIR haplotypes. In addition, a few KIR genes (e.g., 3DS1 and 3DL1) are mutually exclusive since they appear to be allelic variants from a single ancestral locus. These known haplotypes and haplotype patterns can be incorporated into haplotype inference for genotyping data from unrelated individuals.

To effectively use the previously identified haplotypes and haplotype patterns to facilitate the haplotype inference for KIR genes, Yoo et al. (2007) developed a hybrid approach combining a greedy algorithm with the Expectation-Maximization (EM) method. Their method was specifically tailored for haplotype inference of KIR genes and was implemented in a software package, HaploIHP. Their simulation results illustrated that HaploIHP had superior performance compared with two commonly used methods for haplotype inference, the EM-based program HAPLORE (Zhang et al., 2005) and the Hidden Markov Model based method PHASE (Stephens et al., 2001). Their greedy algorithm starts from the set of identified haplotypes to find a minimum number of haplotypes that can resolve the unknown haplotypes pairs for a maximum number of individuals. Then new haplotypes outside the set of identified haplotypes are then added until all individual were solved. After a final set of haplotypes are identified, haplotype frequencies and compatible haplotype pairs with their posterior probabilities for each individuals are estimated by the EM algorithm. In the model of HaploIHP, previously identified haplotypes are used as “true” haplotypes, which will cause problems if some of the identified haplotypes are actually not from the samples under study. Moreover, Fallin and Schork (2000) reported that when the haplotypes are more or less equally frequent, the frequency estimates from EM–based methods can be less accurate.

Recently, several Hidden Markov Model based methods for haplotype inference (Stephens et al., 2001; Scheet and Stephens, 2006; Li et al., 2010) have been developed. Inspired by these methods, we developed a HMM for haplotype inference that can effectively use previously identified haplotypes and haplotype patterns. We evaluated and compared the performance of our method with the method developed by Yoo et al. (2007) based on simulations of KIR gene data from Caucasian populations (Hsu et al., 2002). We also compared the performance of our method with two methods that do not use previously identified haplotypes and haplotype patterns, including an EM based method, HPALORE, and a HMM based method, MaCH.

## Methods

### Notations

We derive a Hidden Markov Model (HMM), called HaploHMM, to improve the haplotype inference using previously identified haplotypes and haplotype patterns. HaploHMM is an extension of the HMM implemented in MaCH, so we will use the same notations as those used in Li et al. (2010).

We denote *N* as the number of diploid samples, *L* as the number of KIR genes under study, *G^n^_l_* as the genotype of *n*-th sample at *l*-th gene. For genotypes composed of present-absent genes, the genotype *G^n^_l_* at locus *l* (*l* = 1, …, *L*) for the individual *n* (*n* = 1, …, *N*) has only three possible alternatives: present, absent, missing. For the absent status, the gene is absent on both chromosomes, and the genotype is represented as (0, 0). For the present status, only one copy is known to be present, and the status of the other chromosome is unknown; in that case, the genotype is represented as (1, ?). For the missing status, the status of both copies is unknown, and the genotype is represented as (?, ?).

### The HMM for haplotype inference in MaCH

For the HMM implemented in MaCH and Thunder (Li et al., 2010), it assumes there is a set reference haplotypes spanning *L* loci. The haplotypes of each individual are imperfect mosaic of those reference haplotypes. We use a series of indicator variables, *S^n^*_1_, …, *S^n^_L_* to represent a hypothetical (and unobserved) state sequence for the individual *n*. At a specific site *l*, diploid state *S^n^_j_* = (*x^n^_j_*, *y^n^_j_*) indicates what the two haplotypes of the individual are and out of the reference haplotypes, respectively.

For the rest of manuscript, we will ignore the superscript since we will build the HMM and infer the underlying mosaic state for each individual separately for a given set of reference haplotypes. The HMM in MaCH (Li et al., 2010) can be described as following:

$$\begin{array}{l} \mathrm{Pr}\text{ ​​​}(G_{1},\;\ldots ,G_{L},\;S_{1},\;\ldots ,\;S_{L})\\ \;\propto \;\mathrm{Pr}\text{​}(S_{1})\;\prod_{j\;=\;2}^{L}\mathrm{Pr}\text{​}(S_{j}|S_{j\;-\;1})\prod_{j\;=\;1}^{L}\mathrm{Pr}\text{​}(G_{j}|S_{j}). \end{array}$$

In the above model, Pr(*S*_1_) is the prior probability of the initial mosaic state and is usually assumed to be equal for all possible compatible haplotype configurations, Pr(*S_l_*|*S*_*l*−1_) denotes the transition probability between two mosaic states and reflects the likelihood of historical recombination events in the interval between *l* and *l* − 1, Pr (*G_l_*|*S_l_*) denotes the emission probability, which is the probability of observed genotypes at each position conditional on the underlying mosaic state and reflects the combined effects of gene conversion, mutation, and genotyping error. Please refer to Li et al. (2010) for the formulas of Pr(*S*_1_), Pr(*S_l_*|*S*_*l*−1_), and Pr(*G_l_*|*S_l_*), which are also used in our HMM model.

The haplotype inference algorithm is basically a Gibbs sampler: a random pair of haplotypes of each individual is assigned according to the observed genotype data. Then, *S*_1_, …, *S_L_* for each individual *n* are sampled separately according to the likelihood function *L*(*S*_1_, …, *S_L_*|*G*_1_, …, *G_L_*) ∝ Pr(*G*_1_, …, *G_L_*, *S*_1_, …, *S_L_*). Specifically, *S_L_* is first sampled according to Pr(*G_L_*, *S_L_*), then *S_l_* (*l* = *L* − 1, …, 1) are sampled according to Pr(*S_l_*|*S*_*l*+1_, …, *S_L_*, *G_l_*). Then *S*_1_, …, *S_L_* are used to impute genotype of that individual according to Pr(*G_l_*|*S_l_*) and determine the new pair of haplotypes of that individual. The sampling procedure is performed over all individuals and repeated for a number of times (e.g., 50–100). The consensus genotype and pair of haplotypes of each individual can then be determined by averaging results over repeats.

### Extended HMM with incorporation of absent-present data, previously identified haplotypes and partial haplotype patterns

Since a KIR gene is either absent or present on a chromosome, there are three possible true genotypes: (0, 0) (both absent), (0, 1) (one absent, one present), and (1, 1) (both present). There are also three observed genotypes from locus-specific genotyping technology: (0, 0), (1, ?), and (?, ?). The incorporation of such missing data in the emission probability in HMM is straightforward: the emission probability is the summation over all possible genotypes that are compatible with the observed genotype. For example, Pr(*G_l_* = (1, ?)|*S_l_*) = Pr(*G_l_* = (1, 0)|*S_l_*) + Pr(*G_l_* = (1, 1)|*S_l_*).

For the present-absent genetic data, the large amount of missing data makes the haplotype inference difficult. However, previously identified haplotypes and haplotype patterns can be used in the HMM to improve the accuracy of haplotype information. Studies so far have revealed 42 different haplotypes involving different combinations of 17 KIR genes (Khakoo and Carrington, 2006), including four—3DL3, 3DP1, 2DL4, and 3DL2—that are found on all KIR haplotypes. In addition, several haplotype patterns have been observed from the empirical data: some genes always appear together on every haplotype, some pairs of genes always appear to be both absent or both present (completely positive LD) on a haplotype, while some pairs of genes never appear together (completely negative LD).

For the HMM for genotype imputation and haplotype, a good choice of reference haplotypes can result in improved accuracy. Although both external haplotypes (e.g., haplotypes obtained from the external reference data such as data from the HapMap Project) and internal haplotypes (haplotypes estimated from individuals in the same study sample) can be used as reference haplotypes (Marchini et al., 2007; Li et al., 2010), studies have shown that using internal haplotypes is very helpful for the accuracy (Zhang et al., 2011). For each iteration of each individual in the HMM, we use internal as well as external reference haplotypes. Here, the internal reference haplotypes refer to haplotypes estimated from other individuals in the same or previous iteration while the external reference haplotypes refer to previously identified haplotypes. It is worth noting that the external reference haplotypes remain same while the internal reference haplotypes change across different iterations and individuals. Such setting allows us to use the information from previously identified haplotypes while avoids the problems from HaploIHP even the previously identified haplotypes are misspecified.

To incorporate previously identified haplotype patterns, we define the following probability function: Pr(*S_l_*, …, *S_L_*|Patterns) = 1 if the haplotype pair determined from *S_l_*, …, *S_L_* are compatible with the haplotype patterns at the sites *l*, …, *L* and Pr(*S_l_*, …, *S_L_*|Patterns) = 0 otherwise. When we perform the backward sampling according to Pr(*S_l_*|*S*_*l*+1_, …, *S_L_*, *G_l_*), we sample *S_l_* such that Pr(*S_l_*, …, *S_L_*|Patterns) = 1. Actually, our sampling is equivalent to sample the hidden sates according to the following likelihood function which is an extension of the likelihood function from the HMM of MaCH (Li et al., 2010):

$$\begin{array}{l} \mathrm{Pr}\text{ ​​​}(G_{1},\;\ldots ,G_{L},\;S_{1},\;\ldots ,\;S_{L},\text{ Patterns})\;\propto \;\mathrm{Pr}\text{​}(S_{1})\prod_{l\;=\;2}^{L}\mathrm{Pr}\text{​}(S_{l}|S_{l\;-\;1})\\ \;\prod_{l\;=\;1}^{L}\mathrm{Pr}\text{​}(S_{l},\;\ldots ,\;S_{L}|\text{Patterns})\prod_{l\;=\;1}^{L}\mathrm{Pr}\text{​}(G_{l}|S_{l}). \end{array}$$

By doing this, we can make sure that the haplotypes obtained from HMM sampling are consistent with the identified haplotype patterns.

### Data simulations

We used the same 17 KIR haplotypes and their frequencies as these in (Yoo et al., 2007) for simulations. These haplotypes and their frequencies are listed in Table 1. To generate the data under the assumption of Hardy-Weinberg Equilibrium (HWE), two haplotypes were randomly selected according to their frequencies and paired to form the genotype of each individual. Then the genotype was converted to the present–absent format. Original haplotype configuration for each individual was stored separately to assess the performance of methods for haplotype inference. Similarly, the data were also generated assuming a departure from HWE by modifying the proportions of heterozygous haplotype pairs and homozygous haplotype pairs in the following way. Let *w_HOM_* be the homozygosity parameter and *w_HET_* be the heterozygosity parameter. *F_HOM_* is the sum of frequencies for all homozygous haplotype pairs and *F_HET_* is the sum of frequencies for all heterozygous haplotype pairs under HWE. We can obtain α that satisfies α(*w_HOM_ F_HOM_* + *w_HET_ F_HET_*) = 1 for given *w_HOM_* and *w_HOM_*. With the HWE assumption, *w_HOM_* and *w_HET_* are set to be equal to 1. We can set *w_HOM_* > *w_HET_* to represent the excessive homozygosity and *w_HOM_* < *w_HET_* to represent the excessive heterozygosity. The new frequency of each haplotype pair with a departure from HWE is obtained by multiplying α*w_HOM_* for homozygous haplotype pairs and α*w_HET_* for heterozygous haplotype pairs. We generated data sets with *w_HOM_* = 1 and *w_HET_* = 2 (excessive heterozygosity) and *w_HOM_* = 2 and *w_HET_* = 1 (excessive homozygosity).

**Table 1:** **17 KIR gene haplotypes with their frequencies used in simulations**.

**Number**	**Haplotypes**	**Frequency**
1	1 0 0 1 0 1 1 0 1 0 0 0 0 1	0.552
2	1 1 1 0 0 0 1 0 1 1 0 0 0 1	0.003
3	1 1 1 0 0 1 1 0 1 1 0 0 0 1	0.015
4	1 0 0 1 0 1 1 1 0 0 0 0 0 1	0.006
5	1 1 1 0 0 0 1 0 1 0 0 0 0 1	0.101
6	1 1 1 0 1 1 1 1 0 1 1 0 1 1	0.037
7	1 1 1 0 0 0 1 1 0 1 0 1 1 1	0.028
8	1 1 1 0 1 1 1 0 1 0 1 0 0 1	0.064
9	1 0 0 1 0 1 1 1 0 1 0 1 1 1	0.107
10	1 0 0 1 1 0 1 1 0 1 1 1 1 1	0.003
11	1 1 1 0 1 1 1 1 0 0 1 0 0 1	0.015
12	1 0 0 1 1 1 1 1 0 0 1 0 1 1	0.018
13	1 0 0 1 0 1 1 0 1 0 0 0 1 1	0.003
14	1 1 1 0 0 1 1 0 1 1 1 0 0 1	0.006
15	1 1 1 0 0 0 1 0 1 1 0 1 1 1	0.006
16	1 1 1 0 0 1 1 0 1 0 0 0 0 1	0.022
17	1 1 1 0 1 0 1 1 0 1 1 1 1 1	0.012

The 14 KIR genes are 3DL3, 2DS2, 2DL2, 2DL3, 2DL5B, 2DL1, 2DL4, 3DS1, 3DL1, 2DL5A, 2DS3, 2DS5, 2DS1, and 3DL2. Among these haplotypes, 10 most frequent haplotypes, 1, 3, 5, 6, 7, 8, 9, 11, 12, and 16 were selected as identified haplotypes in HaploHMM and HaploIHP.

To assess the effect of sample size, we generated data with different sample sizes (50, 100, and 200 individuals). To evaluate the performance of the proposed method under different haplotype frequency distributions, we started with the original frequencies that the most frequent haplotype has the frequency of 55.2%, then gradually decreased the frequency of this major haplotype by 5–10.2%, and increased the frequencies of ten haplotypes with the lowest frequency by 0.5%. To evaluate the performance of the proposed method when some of the identified haplotypes are actually incorrect, we switched alleles of two most frequent haplotypes at two loci. We also generated data sets without missing to compare our method with others. For each setting, we simulated 500 data sets.

### Measures used in comparison of methods for haplotype inference

To quantify the performance of different methods for haplotype inference, we calculated the following four measures for each replicate and take the average of these measures over 500 replicates: an index of performance in terms of haplotype identification (*IH*; Excoffier and Slatkin, 1995), sum of absolute differences between estimated and true frequencies (*SAD*; Fallin and Schork, 2000), individual error rate (*IE*; Niu et al., 2002), and similarity error rate (*SE*; Stephens and Donnelly, 2003). These four measures were selected because they have been extensively used by researchers to evaluate the performance of methods for haplotype inference and each of them provides different aspects of such evaluation. *IH* and *SAD* are computed from the estimated haplotypes and their frequencies, while *IE* and *SE* are computed from haplotype pairs assigned to each individual with their true haplotypes. In order to find where *IE* comes from, we also calculated the other two measures: individual mutation error (*IME*) and individual switch error (*ISE*). *IE* comes from two sources, one is *IME*, which is defined as the proportion of individuals whose haplotypes are assigned incorrectly because the inferred haplotype pairs are not compatible with the genotype; the other one is *ISE*, which is defined as the proportion of individuals whose haplotypes are assigned incorrectly because switching occurred at some loci. If the genotype has no missing data, then *IE* = *ISE*.

Specifically, *IH* is defined as: *IH* = 2(*K_true_* − *K_miss_*)/(*K_true_* + *K_est_*), where *K*_true_ is the number of true haplotypes, *K_est_* is the number of estimated haplotypes, and *K_miss_* is the number of true haplotypes that are not identified. The range of *IH* is from 0 to 1 and the larger value of *IH* indicates the better performance. When the set of estimated haplotypes is the same as the set of true haplotypes, *IH* has the value of 1 indicating the best performance. When the set of estimated haplotypes doesn't contain any true haplotype, *IH* has the value of 0 indicating the worse performance. *SAD* is defined as *SAD* = ∑_*k*_|θ*^est^_k_* − θ*^true^_k_*|. Here θ*^est^_k_* and θ*^true^_k_* are the estimated and true frequency of that haplotype, respectively. *SAD* reflects the overall deviation between the estimated and the true haplotype frequencies. The range of *SAD* is from 0 to 2 and the smaller of *SAD* indicates the better performance. *IE* is defined as the proportion of individuals whose haplotypes were assigned incorrectly while *SE* is defined as the Hamming distance between true and estimated haplotype pairs divided by twice the number of loci. The range of *IE* and *SE* is from 0 to 1 and the smaller values indicate the better performance. The range of *IME* (individual mutation error) and *ISE* (individual switch error) is from 0 to *IE*. In the absence of missing data, *IME* equals to 0 and *ISE* is same as *IE*. Again, the smaller values of *IME* and *ISE* indicate the better performance.

### Identified haplotypes and haplotype patterns

We used 10 most frequent haplotypes in Table 1 as identified haplotypes which were used as reference haplotypes for HaploHMM and as input haplotypes for HaploIHP. To evaluate the performance of the proposed method when some of previously identified haplotypes are actually correct, we switched alleles of two most frequent haplotypes at two loci. Specifically, we switched alleles at loci 2DL5B and 2DL1 for the haplotypes 1 and 9 in Table 1. The frequencies accounted by the identified haplotypes and the incorrect haplotypes were 96% and 66% of total haplotype frequencies when the original haplotype frequencies were used in simulations, respectively. When we gradually decreased the frequency of the most frequent haplotype from 55.2% to 10.2% by 5%, and increased the frequencies of ten haplotypes with the lowest frequency by 0.5% in simulations, the frequencies accounted by the identified haplotypes and the incorrect haplotypes gradually reduced to 65 and 21% of total haplotype frequencies, respectively (Table 2).

**Table 2:** **The different haplotype frequency distributions used in the simulation**.

**Frequency of most frequent haplotype**	**Standard deviation of haplotype frequencies**	**Haplotype frequency accounted by identified haplotypes (%)**	**Haplotype frequency accounted by incorrect haplotypes (%)**
0.552	0.131	96	66
0.502	0.119	93	61
0.452	0.105	89	56
0.402	0.093	86	51
0.352	0.802	82	46
0.302	0.068	79	41
0.252	0.055	75	36
0.202	0.044	72	31
0.152	0.033	68	26
0.102	0.024	65	21

We started with the original frequencies that the most haplotype has the frequency of 55.2%, then gradually decreased the frequency of this major haplotype to 10.2% by 5%, and increased the frequencies of ten haplotypes with the lowest frequency by 0.5%. The corresponding frequency of most frequent haplotype (haplotype 1 in Table 1), the standard deviation, the haplotype frequency accounted by the identified haplotypes and the incorrect haplotypes are listed. It is worth noting that the total of 17 haplotypes were used in the simulation and the summation of haplotype frequencies is equal to 1, thus the standard deviation times 17 is the coefficient of variation (CV).

We used three types of haplotype patterns derived from the observations in KIR haplotype studies (Hsu et al., 2002; Marsh et al., 2003; Middleton et al., 2005): (1) three anchor genes, 2DL4, 3DL2 and 3DL3 always present in all haplotypes; (2) two genes, 2DS2 and 2DL2, always either present or absent together in all haplotypes and (3) two pairs of genes, (3DS1, 3DL1) and (2DL2, 2DL3), in complete negative LD, i.e., when one gene in each pair is present in a haplotype, the other gene in the same pair is absent.

## Results

Yoo et al. (2007) showed by simulation that HaploIHP is better than PHASE (Stephens et al., 2001) and HAPLORE (Zhang et al., 2005) for KIR data even when 60 and 25% of previously identified haplotypes were incorporated into the analysis. Here we compared the performance of our method with HaploIHP (Yoo et al., 2007), HAPLORE (Zhang et al., 2005), and MaCH (Li et al., 2010). We evaluated their performances by all six measures (*IH*, *SAD*, *IE*, *IME*, *ISE*, and *SE*).

**Figure 1:**
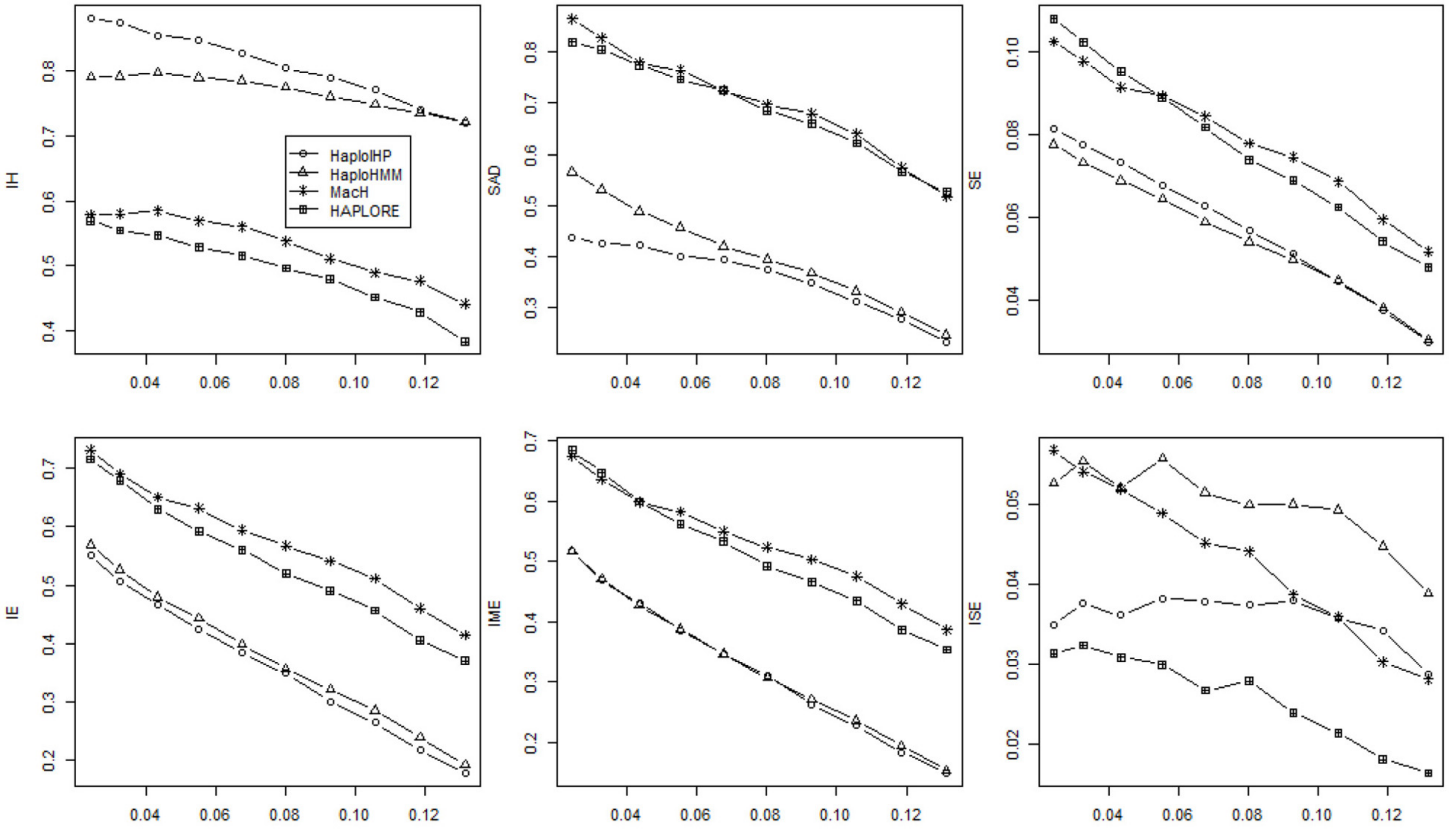
**Average values of six measures (*IH*, *SAD*, *SE*, *IE*, *IME*, and *ISE*) over 500 replicates with the sample size of 100 and the assumption of HWE under different haplotype frequency distributions**. The x-axis represents the standard deviation of haplotype frequencies used in simulations. Results were obtained when no incorrect haplotypes were included as identified haplotypes.

**Figure 2:**
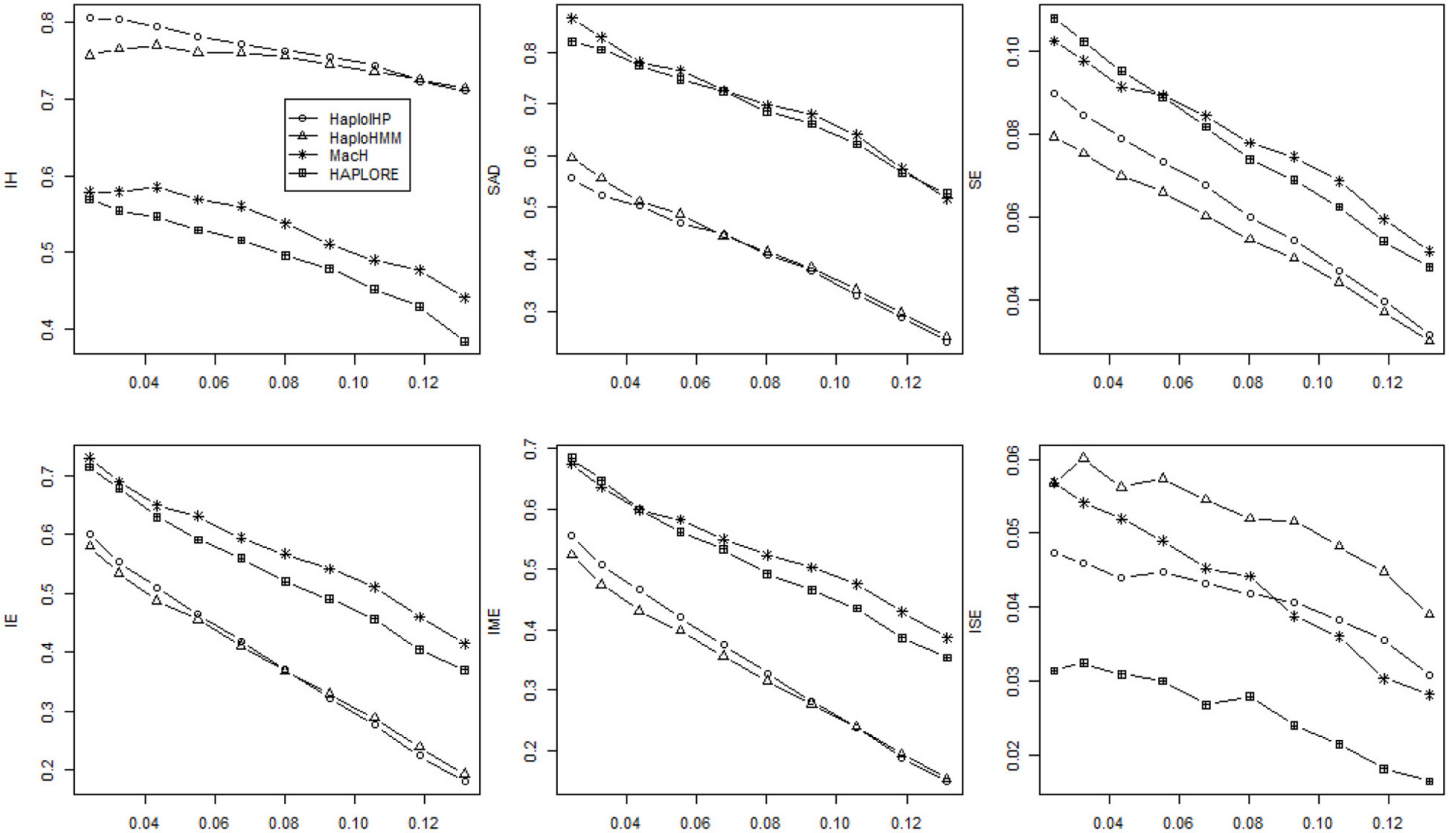
**Average values of six measures (*IH*, *SAD*, *SE*, *IE*, *IME*, and *ISE*) over 500 replicates with the sample size of 100 and the assumption of HWE under different haplotype frequency distributions**. The x-axis represents the standard deviation of haplotype frequencies used in simulations. Results were obtained when two incorrect haplotypes were included as identified haplotypes.

Figures 1, 2 show the average values of six measures over 500 replicates with the sample size of 100 and the assumption of HWE under different haplotype frequency distributions (Table 2). It can be seen that the average *IH* values from HaploHMM and HaploIHP ranged from 0.72 to 0.88 and were much higher for the average values from MaCH and HAPLORE which ranged from 0.38 to 0.57. The average *IH* values decreased slightly with the increasing of standard deviation of haplotype frequencies used in simulations. When the correct haplotypes were used as identified haplotypes, HaploIHP always had better performance than HaploHMM. When some incorrect haplotypes were included as identified haplotypes, HaploHMM had the slightly larger *IH* values when the standard deviation of haplotype frequencies was larger than 0.11. It is worth noting that the EM based method (HaploIHP) had the larger *IH* values than those of the HMM based greedy method (HaploHMM) when the identified haplotypes and haplotype patterns were used while the EM based method (HAPLORE) had the smaller *IH* values than those of the HMM based method (MaCH) when the identified haplotypes and haplotype patterns were used. This is because that HaploIHP uses the identified haplotypes and haplotype patterns to reduce the number of compatible haplotypes in the EM thus results in more accurate estimation of haplotypes, HAPLORE results in many haplotypes with small frequency due to a large number of compatible haplotypes from the missing data thus has the smaller *IH* values. In terms of *SAD*, the sum of differences between true haplotype frequencies and estimated haplotype frequencies from HaploHMM ranged from 0.25 to 0.57 and were always bigger than those from HaploIHP. This is not unexpected since HaploHMM only used 200 haplotypes from the last round of HMM iteration to estimate haplotype frequencies while HaploIHP used the EM algorithm. The average values of *SAD* from HaploIHP and HaploHMM were smaller than those from MaCH and HAPLORE.

As shown in Figures 1, 2, both *IE* and *SE* decreased when the standard deviation of haplotype frequencies used in simulations increased, indicating that all methods performed better if there were one or a few major haplotypes. The change of *IE* and *SE* were much larger than *IH* and *SAD*. For HaploHMM, the average *IE* reduced from 0.58 to 0.19 while *SE* reduced from 0.08 to 0.30. In terms of *SE*, HaploHMM always had the smaller *SE* than those of HaploIHP, indicating better performance of HaploHMM. In terms of *IE*, HaploIHP had better performance than HaplloHMM when the correct haplotypes were used as identified haplotypes while HaploHMM had better performance than HaploIHP when the standard deviation of haplotype frequencies was smaller than 0.08 and the incorrect haplotypes were included in identified haplotypes To further investigate *IE*, we distinguished two types of error: *IME* and *ISE* and presented the results in Figures 1, 2. It can be seen that the majority of *IE* was from *IME*. *IME* showed similar patterns with *IE*: HaploHMM had the smaller *IME* when the standard deviation of haplotype frequencies is small while HaploIHP had the smaller *IME* when the standard deviation of haplotype frequencies is large. Both HaploIHP and HaploHMM had the smaller SE and IE than those of MaCH and HAPLORE, indicating the use of identified haplotypes and haplotype patters significantly improved the accuracy for haplotype inference.

**Figure 3:**
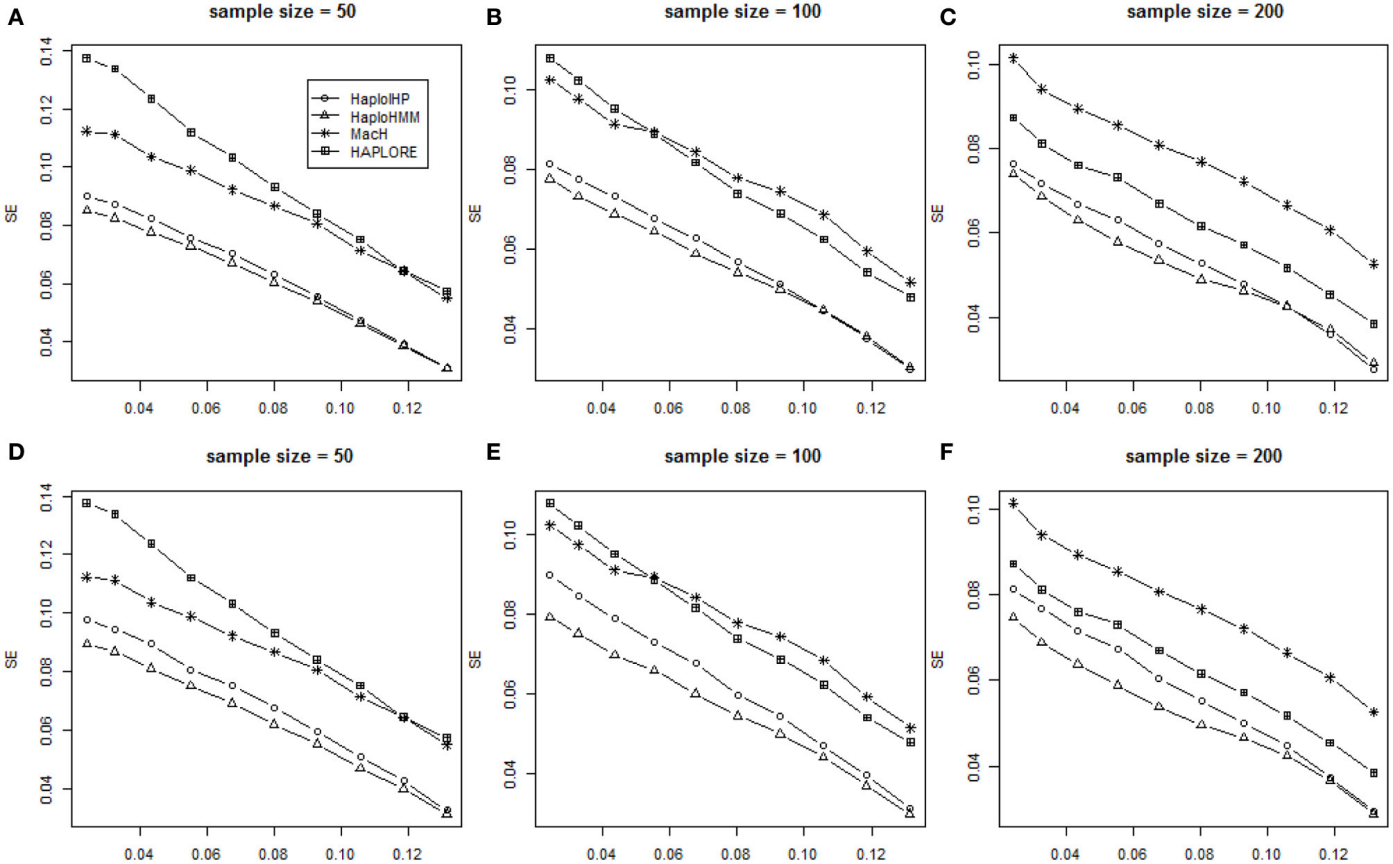
**Average values of SE with the sample size of 50, 100, and 200 and the assumption of HWE under different haplotype frequency distributions**. The x-axis represents the standard deviation of haplotype frequencies used in simulations. **(A–C)** represent the results when no incorrect haplotypes were included as identified haplotypes while **(D–F)** represent the results when some incorrect haplotypes were included as identified haplotypes.

We assessed the performance of HaploHMM and HaploIHP with different sample size of 50, 100, and 200. Patterns of six measures from HaploHMM, HaploIHP, MaCH, and HAPLORE with the sample size of 50 and 200 were similar as those with the sample size of 100. Figure 3 shows the average *SE* values for measures with the sample size of 50, 100, and 200. It can be seen that HaploHMM outperformed HaploIHP and HaoloHMM and HaploIHP had much better performance than MaCH and HAPLORE in terms of *SE*. For both HaploHMM and HaploIHP, the effect of sample size is rather smaller, suggesting that increasing sample size from 50 to 200 does not significantly improve the accuracy for haplotype inference.

**Figure 4:**
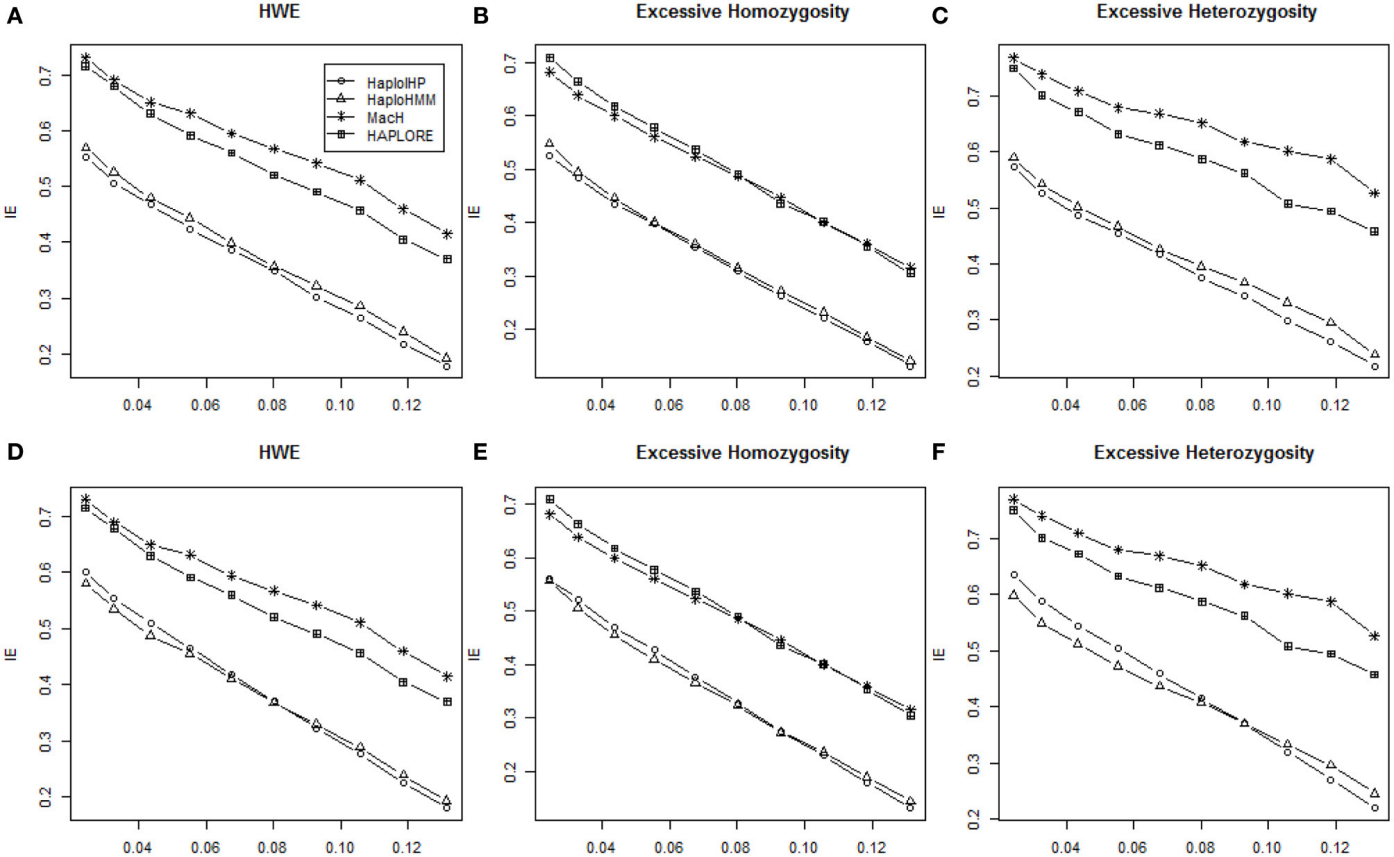
**Average values of IE with the sample size of 100 and with HWE, excessive heterozygosity, and excessive homozygosity**. The x-axis represents the standard deviation of haplotype frequencies used in simulations. **(A–C)** Represent the results when no incorrect haplotypes were included as identified haplotypes while **(D–F)** represent the results when some incorrect haplotypes were included as identified haplotypes.

We investigated the effect of departure from HWE on the performance of the four methods using simulated data with HWE, excessive homozygosity, and excessive heterozygosity. Again, patterns of six measures from HaploHMM and HaploIHP with excessive homozygosity and excessive heterozygosity were similar as those with the assumption of HWE. It is expected that both methods had better performance with excessive homozygosity while worse performance with excessive heterozygosity due to the reduced haplotype ambiguity with excessive homozygosity. Figure 4 shows the average *IE* measures for different situations. The *IE* decreased as standard deviation of haplotype frequencies increased in most situations. When only correct haplotypes were included as identified haplotypes, HaploIHP had a lower *IE* value (thus better performance) than those of HaploHMM. However, when some incorrect haplotypes were included as identified haplotypes, HaploHMM had lower *IE* values than those of HaploIHP, except a few cases when standard deviation of haplotype frequencies was quite large. Again, HaoloHMM and HaploIHP had much better performance than MaCH and HAPLORE.

**Figure 5:**
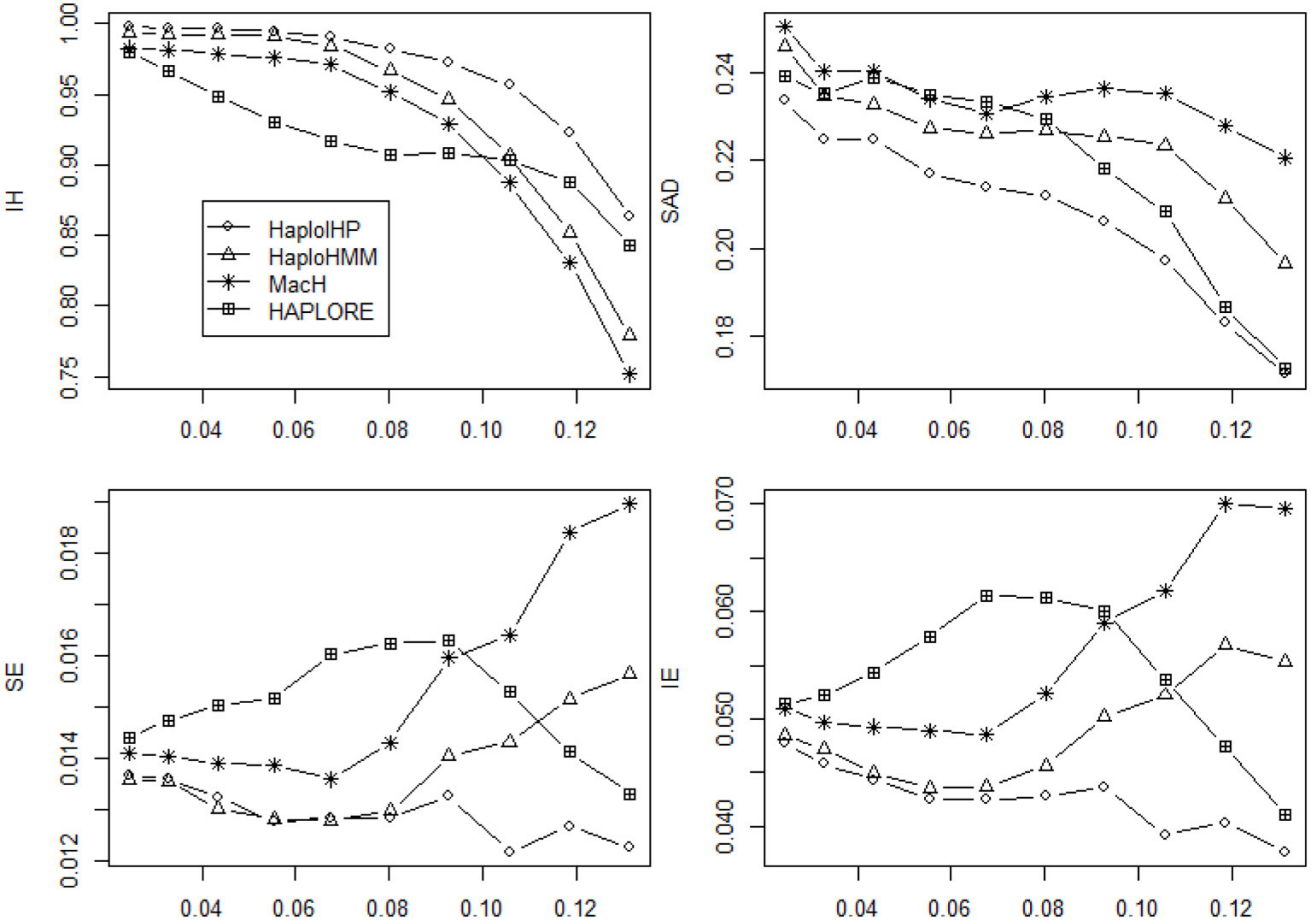
**Average values of six measures (*IH*, *SAD*, *SE*, and *IE*) over 500 replicates with the sample size of 100 and the assumption of HWE under different haplotype frequency distributions**. The x-axis represents the standard deviation of haplotype frequencies used in simulations. Results were obtained when there was no missing data and there was no incorrect haplotypes included as identified haplotypes.

**Figure 6:**
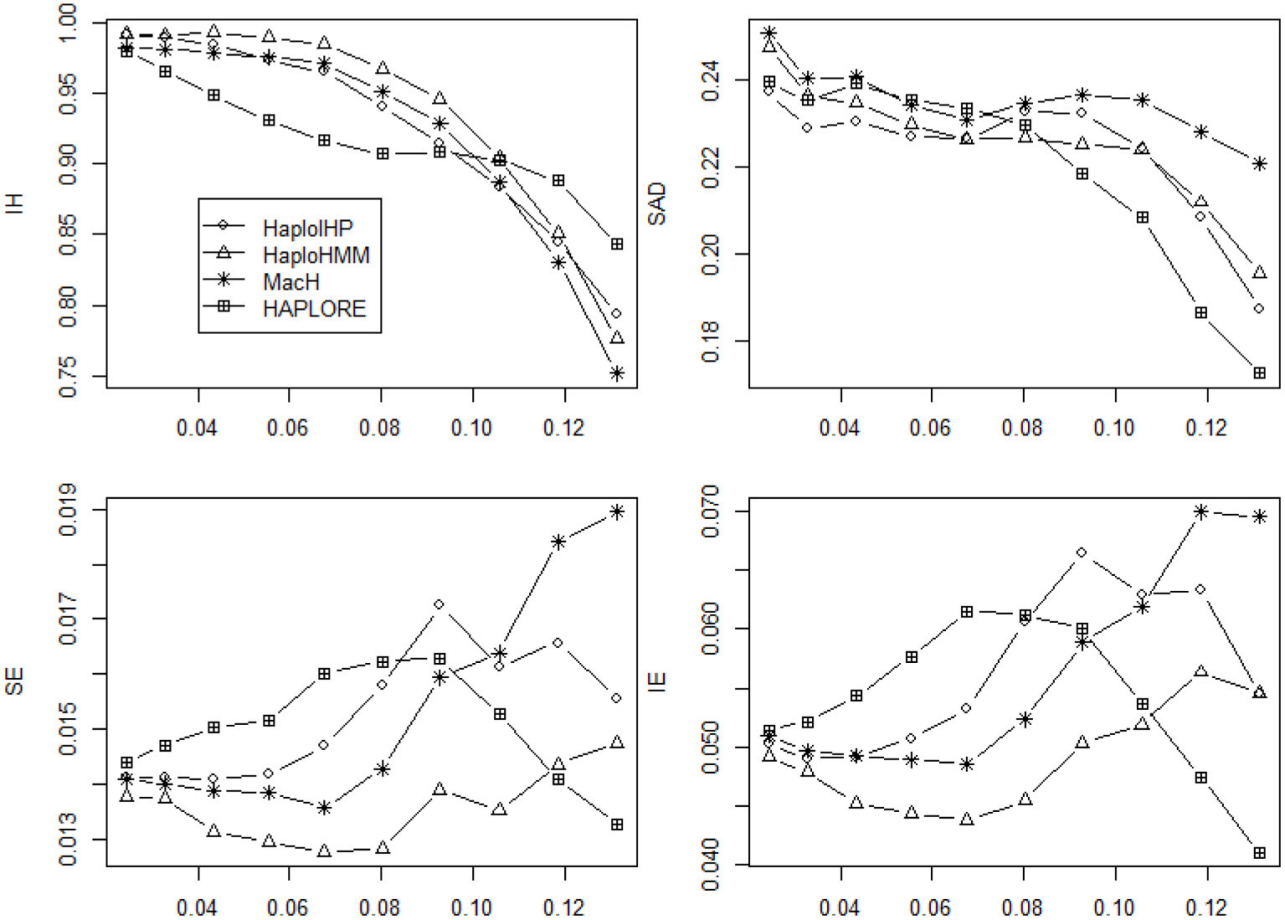
**Average values of six measures (*IH*, *SAD*, *SE*, and *IE*) over 500 replicates with the sample size of 100 and the assumption of HWE under different haplotype frequency distributions**. The x-axis represents the standard deviation of haplotype frequencies used in simulations. Results were obtained Results were obtained when there was no missing data and there were two incorrect haplotypes included as identified haplotypes.

We investigated if the use of identified haplotypes and haplotype patterns can improve the accuracy for haplotype inference in the absence of missing data and presented the average values of four measures (*IH*, *SAD*, *SE*, and *IE*) in Figures 5, 6. In the absence of missing data, all methods had much better performance and the differences of the average values of four measures among four methods were much smaller than those of in the presence of missing data. HAPLORE had the best performance when the standard deviation of haplotype frequency was large while HAPLORE had the worst performance when the standard deviation of haplotype frequency was large. When only correct haplotypes were included as identified haplotypes, HaploIHP still had the best performance, followed by HaploHMM and MaCH. However, when some incorrect haplotypes were included as identified haplotypes, HaploHMM had the best performance across all haplotype frequency distributions and MaCH outperformed HaploIHP when the standard deviation of haplotype frequency was less than 0.10.

## Discussion

Many methods for haplotype inference have been developed and widely used by researchers. Although these methods can be directly applied to present-absent data (Liu et al., 2006), the large portion of missing data can greatly affect their accuracy for haplotype inference. In addition, most of these methods have not incorporated identified haplotypes and/or haplotype patterns for improved accuracy. In this paper, we developed a Hidden Markov Model that incorporate identified haplotypes and/or haplotype patterns for haplotype inference and illustrated evidence that our HMM can improve the accuracy for the inference of KIR gene haplotype. When compared with HaploIHP, a publically available program that is specially tailored for haplotype inference of KIR genes through simulations, our method, HaploHMM had the better performance when some incorrect haplotypes were included as identified haplotypes and/or the standard deviation of haplotype frequencies were small. Both HaploHMM and HaploIHP had the better performance than MaCH and HAPLORE in the presence of large portion of missing data, indicating the use of identified haplotypes and haplotype patterns can significantly improve the accuracy for haplotype inference in such situation. In the absence of missing data, HaploHMM still had the better performance than HaploIHP when some incorrect haplotypes were included.

If some studies have been conducted for the similar population samples, the identified haplotypes from these studies are likely to be observed again. It is expected that the incorporation of these haplotypes in our program will improve the accuracy for haplotype inference. The use of these haplotypes can especially benefit present-absent genotype data since many individuals may have a large number of compatible haplotype pairs due to the large portion of missing data. The incorporation of such identified haplotypes is straightforward in the HMM—these haplotypes are added to the reference haplotypes (external reference haplotypes). Actually, it has been illustrated that the use of reference haplotypes, such as haplotypes from the HapMap project and the 1000 Genomes Project can improve accuracy for genotype imputation and haplotype inference. The sampling nature of the HMM implemented in HaploHMM also avoids the problem of HaploIHP when some identified haplotypes are misspecified. We calculated the haplotype frequency accounted by incorrect haplotypes that were included as identified haplotypes and used it to measure the degree of incorrectness. We found not only the degree of incorrectness but also the standard deviation of haplotype frequency affected the performance of the performance of HaploIHP and HaploHMM. When there were a very few number of major haplotypes, HaploIHP using the correct haplotypes as identified haplotypes had comparable performance with HaploIHP using the incorrect haplotypes as identified haplotypes, even the incorrect haplotypes accounted a high portion of total haplotype frequency. However, when there were a number of major haplotypes, HaploIHP using the correct haplotypes as identified haplotypes had moderately better performance with HaploIHP using the incorrect haplotypes as identified haplotypes, even the incorrect haplotypes accounted a much lower portion of total haplotype frequency. In contrast, the performance of HaploHMM was much less affected by including the incorrect haplotypes as reference haplotypes.

The set of haplotype patterns can also eliminate haplotypes unlikely observed, so the use of them can improve the efficiency and accuracy for haplotype inference. In our HMM, we incorporate these haplotype patterns by sampling the hidden states that only consistent with haplotype patterns. One drawback of the use of these constrains is that some individuals may not have compatible haplotype pairs compatible with these constraints. In this situation we can sample the hidden states with higher probability if they are consistent with haplotype patterns and with much lower probability if they are not consistent with haplotype patterns. This can be done by defining anew probability function: Pr(*S_l_*, …, *S_L_*|Patterns) = 1 − β if the haplotype pair determined from *S_l_*, …, *S_L_* are compatible with the haplotype patterns at the site *l*, …, *L* and Pr(*S_l_*, …, *S_L_*|Patterns) = β otherwise, where β is small positive number. This allows for the identification of novel haplotypes not seen in the reference haplotypes.

Throughout the paper, we used data from KIR genes as an illustration. Our method can be directly applied to present-absent data from other genes, such as Human Leukocyte Antigen (HLA) motifs determined by sequence specific oligonucleotide assays (Song et al., 2009). In addition, our simulation results illustrated the use of identified haplotypes and haplotype patterns can improve the accuracy of haplotype inference even in the absence of missing data, therefore our method can be used for haplotype inference in general situations.

### Conflict of interest statement

The authors declare that the research was conducted in the absence of any commercial or financial relationships that could be construed as a potential conflict of interest.
